# Fabrication of functional hollow microspheres constructed from MOF shells: Promising drug delivery systems with high loading capacity and targeted transport

**DOI:** 10.1038/srep37705

**Published:** 2016-11-23

**Authors:** Xuechuan Gao, Xiao Hai, Huricha Baigude, Weihua Guan, Zhiliang Liu

**Affiliations:** 1 College of Chemistry and Chemical Engineering, Inner Mongolia University, Hohhot, 010021, P.R. China

## Abstract

An advanced multifunctional, hollow metal-organic framework (MOF) drug delivery system with a high drug loading level and targeted delivery was designed and fabricated for the first time and applied to inhibit tumour cell growth. This hollow MOF targeting drug delivery system was prepared via a simple post-synthetic surface modification procedure, starting from hollow ZIF-8 successfully obtained for the first time via a mild phase transformation under solvothermal conditions. As a result, the hollow ZIF-8 exhibits a higher loading capacity for the model anticancer drug 5-fluorouracil (5-FU). Subsequently, 5-FU-loaded ZIF-8 was encapsulated into polymer layers (FA-CHI-5-FAM) with three components: a chitosan (CHI) backbone, the imaging agent 5-carboxyfluorescein (5-FAM), and the targeting reagent folic acid (FA). Thus, an advanced drug delivery system, ZIF-8/5-FU@FA-CHI-5-FAM, was fabricated. A cell imaging assay demonstrated that ZIF-8/5-FU@FA-CHI-5-FAM could target and be taken up by MGC-803 cells. Furthermore, the as-prepared ZIF-8/5-FU@FA-CHI-5-FAM exhibited stronger cell growth inhibitory effects on MGC-803 cells because of the release of 5-FU, as confirmed by a cell viability assay. In addition, a drug release experiment *in vitro* indicated that ZIF-8/5-FU@FA-CHI-5-FAM exhibited high loading capacity (51%) and a sustained drug release behaviour. Therefore, ZIF-8/5-FU@FA-CHI-5-FAM could provide targeted drug transportation, imaging tracking and localized sustained release.

Metal-organic frameworks (MOFs), which are self-assembled from various metal ions as nodes and organic linkers, have garnered broad attention because of their attractive properties, which include permanent ultra-high porosity, a large specific surface area, a tunable shape, a stable structure and versatile functionality^1^,^2^,^3^,^4^. The nature of MOFs generally imparts them with excellent biodegradability, low cytotoxicity and versatile functionality, thus providing ideal candidates as drug delivery hosts^5^,^6^. Since Férey and co-workers first demonstrated that MOFs can adsorb and deliver drugs efficiently^7^, researchers have made some advances in MOFs as drug delivery systems. Horcajada and co-workers have successfully adopted Fe-based MOFs as a category of biomedical materials for drug delivery^8^. Su and co-workers reported an anionic Zn-based MOF that exhibited a remarkable capacity for cationic drug hosting and controlled delivery^9^. Devic and co-workers prepared a biocompatible porous Mg-based MOF under environmentally friendly conditions as an antioxidant carrier^10^. Although these MOFs exhibit drug hosting and controlled delivery capability, they are not capable of the targeted delivery of drug molecules, resulting in significant toxicity to normal cells and limiting their biomedical applications. Another drawback is the small pore size of MOFs, which limits their drug loading efficiency. To overcome this problem, the fabrication of hollow MOF microspheres that possess deep submicron-scale cavities and MOF shells with intrinsic pores as windows, together with folic acid (FA) as the targeting agent on the microsphere surface, has been achieved; the resulting MOF microspheres are a promising drug delivery system for cancer treatment.

Zeolitic imidazolate frameworks (ZIFs), an important subclass of MOFs, are known for their nontoxic nature and unusually large available voids^11^,^12^. Since the discovery of ZIFs, they have emerged as ideal candidates and have been studied extensively for biomedical applications^13^,^14^,^15^. Among ZIF materials, ZIF-8 is the most frequently studied because of its exceptional chemical and thermal stability^16^,^17^. ZIF-8, which is composed of Zn and 2-methylimidazole, has sufficiently large pore apertures for passing drug molecules. In addition, hollow particles are fascinating materials because of their high drug loading capability^18^,^19^. Thus, hollow ZIF-8 with tunable functionality and controllable morphology fulfils the pre-requisites for an outstanding drug carrier.

To enhance an anticancer drug’s therapeutic effects, a drug carrier should be bonded with specific molecules that can distinguish cancer cells from normal cells. FA is a suitable targeting molecule because the folate receptor is often over expressed on many cancer cell surfaces and has been rarely observed on normal cell surfaces^20^,^21^. Moreover, FA possesses high selectivity and binding affinity to the folate receptor, which has led to wide studies of FA as a stable and effective target-specific agent^22^,^23^. However, directly modifying the surface of ZIF-8 with FA is difficult. A chitosan (CHI) coating can serve as a good option to overcome this barrier. CHI contains many amino and hydroxyl groups and therefore has been used extensively for coupling with other functional groups^24^,^25^. Furthermore, nanomaterials can generate hydrogen bonds with CHI molecules through surface hydroxyls when inorganic nanomaterials are dispersed into CHI solutions^26^. Meanwhile, CHI does not induce immune rejections or allergic reactions, making it promising for enhancing the solubility, permeability and stability of inorganic nanomaterials^27^,^28^. In addition, a CHI coating can prevent quick recognition and elimination of inorganic nanomaterials by the immune system, thereby prolonging their circulation in the body^29^. Because it exhibits such properties, CHI is regarded as a sustainable material for the functionalization of inorganic materials.

Moreover, fluorescent probes are a powerful tool for the real-time visualization and tracking of drugs in living cells. The peripheral carboxyl on 5-carboxylfluorescein (5-FAM) can be easily immobilized onto the CHI, meaning that 5-FAM has the potential to be used as a fluorescent probe for monitoring the drug delivery process^30^. Thus, in this paper, a multifunctional drug delivery system for specific targeted drug delivery and fluorescence tracking imaging is reported; our objective is to minimize side effects and improve the efficiency of cancer treatment (Fig. 1). Herein, we demonstrate the fabrication of hollow MOF microparticles via a one-step solvothermal reaction of Zn(NO_3_)_2_ and 2-methylimidazole without extra template materials. FA-CHI-5-FAM is prepared by bonding the fluorescent molecule 5-FAM and targeting molecule FA to CHI through a reaction between the carboxyl groups of FA, 5-FAM and the amino groups of the CHI chain. 5-Fluorouracil (5-FU), an antimetabolite compound that can prevent tumour-cell pyrimidine nucleotide synthesis, is used as a model drug. Subsequently, hollow ZIF-8-loaded with 5-FU is coated with FA-CHI-5-FAM, resulting in the formation of ZIF-8/5-FU@FA-CHI-5-FAM. We observe that ZIF-8/5-FU@FA-CHI-5-FAM has a high drug loading level (51%). Although the drug loading capacity of ZIF-8/5-FU@FA-CHI-5-FAM does not exceed the carrier-free drug nanoparticles^31^,^32^,^33^, this system is superior to most drug carriers. Meanwhile they can enhance an anticancer drug’s therapeutic effects by targeted drug delivery and fluorescence imaging, making them more effective than carrier-free drug nanoparticles. In addition, compared with most of the existing pure organic and inorganic carrier materials, the ZIF-8/5-FU@FA-CHI-5-FAM is not stable in acidic condition, providing excellent biodegradability and low cytotoxicity as drug delivery hosts. All in all, this system can be used to provide a sustained drug release while allowing the targeting of cancer cells and cellular imaging. Furthermore, such systems, which can function in the targeting, imaging, and therapy domains, have the potential to overcome the conventional limitations of cancer diagnosis and therapy.

## Results and Discussion

### Fabrication and Characterization of ZIF-8/5-FU@FA-CHI-5-FAM

**Figure 2:**
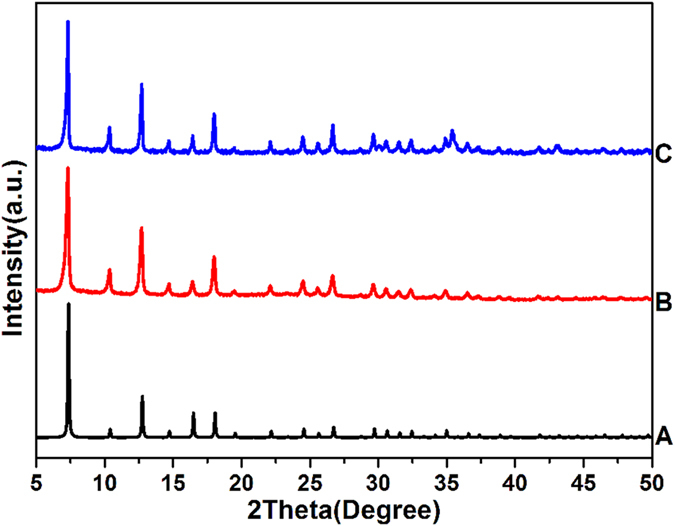
XRD patterns of simulated ZIF-8 (**A**), as-synthesized hollow ZIF-8 (**B**) and ZIF-8/5-FU@FA-CHI-5-FAM (**C**).

The crystal structures of the obtained nanocrystals are studied by PXRD to verify the formation of the desired structure of ZIF-8. As depicted in Fig. 2, the XRD patterns of as-synthesized hollow ZIF-8 (Fig. 2B) and ZIF-8/5-FU@FA-CHI-5-FAM (Fig. 2C) correspond well with the simulated pattern (Fig. 2A). No impurity peaks are observed, suggesting that the ZIF-8 is well crystallized and that the intercalation of 5-FU as well as the encapsulation of FA-CHI-5-FAM only minimally impact the crystalline integrity of the ZIF-8.

**Figure 3:**
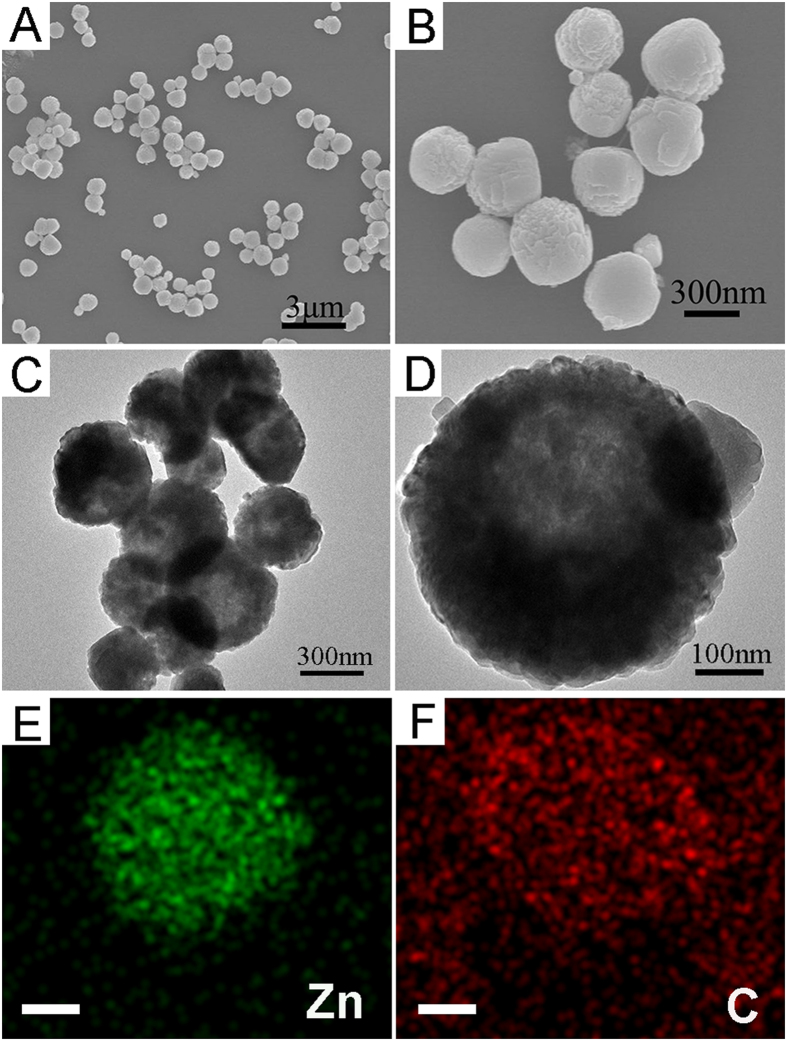
SEM images of ZIF-8/5-FU@FA-CHI-5-FAM with different magnifications (**A,B**); TEM images of ZIF-8/5-FU@FA-CHI-5-FAM with different magnifications (**C,D**); The EDS elemental mapping results of Zn, C in ZIF-8/5-FU@FA-CHI-5-FAM composite (**E,F**). Scale bar: 100 nm.

As shown by the SEM images in Fig. 3A,B, the obtained ZIF-8/5-FU@FA-CHI-5-FAM exhibits a well-defined spherical morphology with a narrow size distribution of approximately 400 nm. The TEM images in Fig. 3C,D demonstrate that the ZIF-8/5-FU@FA-CHI-5-FAM has a distinctive hollow nanocage compared with the solid ZIF-8 shown in Supplementary Figure S1. In addition, EDS mapping of the elemental analysis confirms the existence of an FA-CHI-5-FAM shell, as shown in Fig. 3E,F. The Zn is distributed at the interior of the frame structure, whereas the distribution of C exceeds the frame structure. Furthermore, confocal laser scanning microscopy images of ZIF-8/5-FU@FA-CHI-5-FAM (Supplementary Figure S2) show strong green luminescent signal, originated from the characteristic fluorescence of 5-FAM, suggesting the successful coating of FA-CHI-5-FAM. After dynamic light scattering characterization (Supplementary Figure S3), the average diameter of hollow ZIF-8 is found to be 408 nm (PDI = 0.044). As a comparison, the ZIF-8/5-FU@FA-CHI-5-FAM has a diameter of 415 nm (PDI = 0.043). These measurements indicate that coating FA-CHI-5-FAM onto preformed ZIF-8 does not lead to any signficant change in hydrodynamic diameter, demonstrating the small content of FA-CHI-5-FAM.

**Figure 4:**
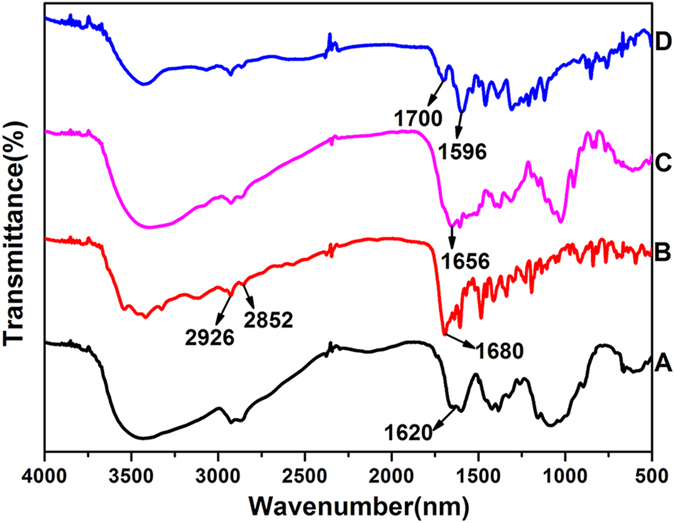
FTIR spectra of CHI (**A**), FA (**B**), 5-FAM (**C**) and FA-CHI-5-FAM (**D**).

**Figure 5:**
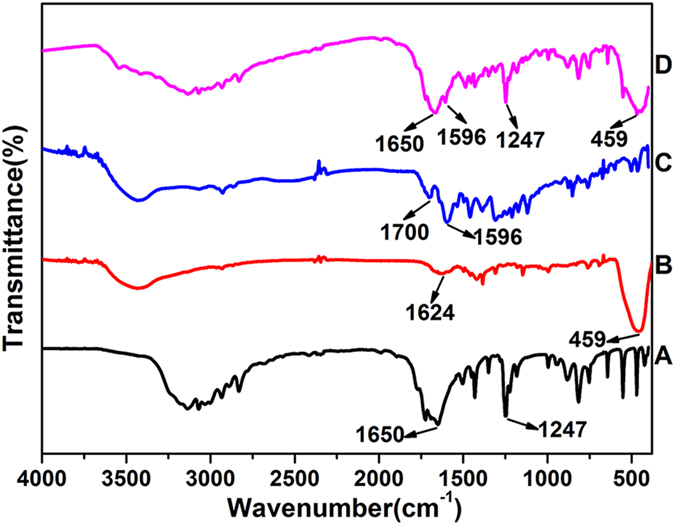
FTIR spectra of 5-FU (**A**), hollow ZIF-8 (**B**), FA-CHI-5-FAM (**C**) and ZIF-8/5-FU@FA-CHI-5-FAM (**D**).

The formation of FA-CHI-5-FAM and ZIF-8/5-FU@FA-CHI-5-FAM are confirmed by FTIR analysis. As shown in Fig. 4, the FTIR spectra of CHI, FA, 5-FAM and FA-CHI-5-FAM all exhibit characteristic peaks at 2926 cm^−1^ and 2852 cm^−1^, which correspond to C-H vibrations. The broad band from 3650 to 3300 cm^−1^ is assigned to N-H stretching vibrations and the peak at 1620 cm^−1^ is associated with the N-H symmetrical deformation mode in the FTIR spectra of CHI (Fig. 4A). In the FTIR spectra of FA (Fig. 4B) and 5-FAM (Fig. 4C), the band from 3650 to 3300 cm^−1^ is due to O-H stretching vibrations and characteristic peaks that appear at 1680 cm^−1^ and 1665 cm^−1^ are assigned to the stretching vibrations of the C=O of carboxyl groups. Compared with the spectra in Fig. 4A–C, the FTIR spectrum of FA-CHI-5-FAM conjugates shows characteristic bands of CHI, FA and 5-FAM, as depicted in Fig. 4D. In addition, two new peaks from the amide I and amide II bands emerge at 1700 cm^−1^ and 1596 cm^−1^, suggesting the formation of amide bonds through a reaction between the COOH of FA, 5-FAM and the NH_2_ of the CHI chain. This result demonstrates that FA-CHI-5-FAM is successfully prepared; its chemical structure is shown in Supplementary Figure S4. Additionally, the Uv-vis absorption spectrum of FA-CHI-5-FAM (Supplementary Figure S5D) further confirms this results. The Uv-vis absorption spectra of 5-FAM (Supplementary Figure S5A), CHI (Supplementary Figure S5B) and FA (Supplementary Figure S5C) show absorption peaks at 470 nm, 300 nm and 206 nm, respectively. And the FA-CHI-5-FAM display all absorption peaks of 5-FAM, CHI and FA.

Figure 5 represents the FTIR spectra of 5-FU, hollow ZIF-8, FA-CHI-5-FAM and ZIF-8/5-FU@FA-CHI-5-FAM. The peak at 1650 cm^−1^ is attributed to the vibrations of C=O in 5-FU (Fig. 5A). The broad band at 1624 cm^−1^ is assigned to the C = C vibration absorption in ZIF-8 (Fig. 5B). Two sharp absorption peaks at 1700 and 1596 cm^−1^ in Fig. 5C correspond to the vibrations of the amide I and amide II bands. As shown in Fig. 5D, the FTIR spectra of ZIF-8/5-FU@FA-CHI-5-FAM display all the prominent peaks of 5-FU, hollow ZIF-8, and FA-CHI-5-FAM stretching vibrations, indicating the successful fabrication of ZIF-8/5-FU@FA-CHI-5-FAM. The peaks at 1650 cm^−1^ and 1247 cm^−1^ have a correlation with 5-FU. While the peaks at 459 cm^−1^ and 1596 cm^−1^ are related to hollow ZIF-8 and FA-CHI-5-FAM, respectively. Compared with FA-CHI-5-FAM and ZIF-8, the peak around 3500 cm^−1^, derived from the N-H stretching vibration in ZIF-8/5-FU@FA-CHI-5-FAM, becomes broaden and red-shifted, indicating the formation of hydrogen bonds between the NH_2_ on the surface of ZIF-8 and NH_2_ on CHI. That is to say, the successful coating of FA-CHI-5-FAM on the surface of ZIF-8 is obtained via the hydrogen bonds between ZIF-8 and CHI. The formation of ZIF-8/5-FU@FA-CHI-5-FAM is further examined by TGA experiments. TGA curves of hollow ZIF-8 and ZIF-8/5-FU@FA-CHI-5-FAM are shown in Supplementary Figure S6. The frameworks of hollow ZIF-8 begin to decompose when the temperature exceeds 600 °C, whereas ZIF-8/5-FU@FA-CHI-5-FAM begins to lose weight at approximately 250 °C because of decomposition of the loaded 5-FU and the FA-CHI-5-FAM coating. Calculating by the TGA data and UV-vis absorption spectra, the contents of FA, 5-FAM and CHI in the as-synthesized sample are 1.5%, 0.8% and 7.7%, respectively. And the standard concentration curve of FA and 5-FAM in aqueous solution are shown in Supplementary Figures S7 and S8.

### Evaluation and Characterization of Cancer Targeting

**Figure 6:**
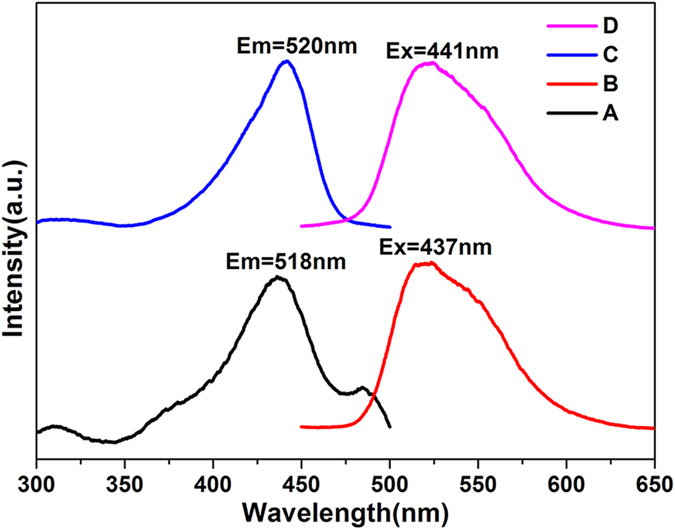
The excitation (**A**) and emission (**B**) spectra of 5-FAM and the excitation (**C**) and emission (**D**) spectra of ZIF-8/5-FU@FA-CHI-5-FAM.

**Figure 7:**
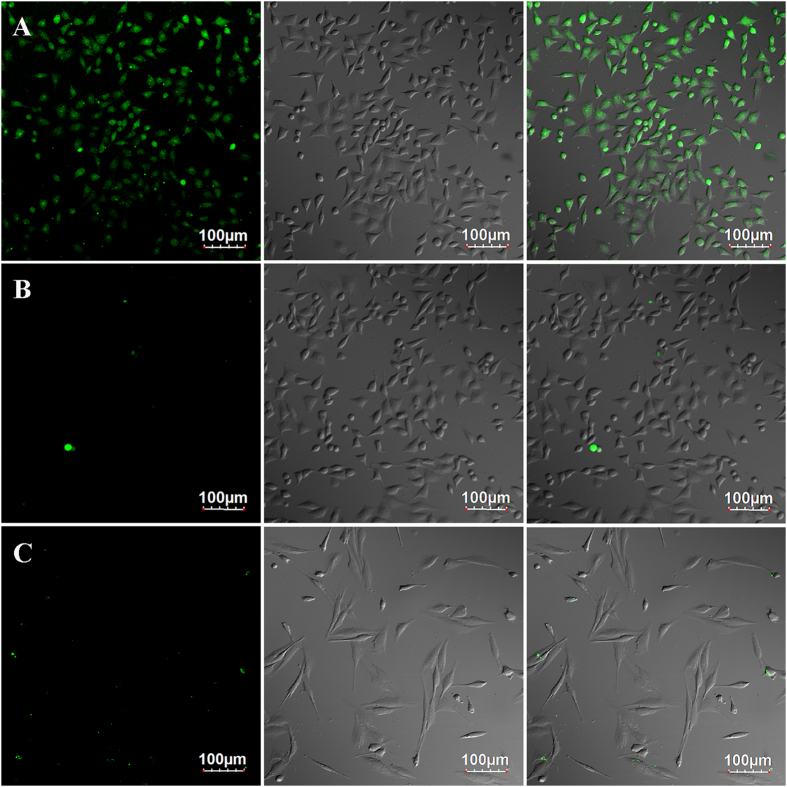
Fluorescence imaging of live MGC-803 cells after being incubated with ZIF-8/5-FU@FA-CHI-5-FAM (**A**) and ZIF-8/5-FU@ CHI-5-FAM (**B**) nanomaterials for 2 h. Fluorescence imaging of live HASMC cells after being incubated with ZIF-8/5-FU@FA-CHI-5-FAM (**C**) nanomaterials for 2 h. The left panels show dark-field fluorescence images, the middle panels show the corresponding bright-field images and the right panels are overlays of the left and middle panels. Scale bar: 100 μm.

The photoluminescence properties of 5-FAM and ZIF-8/5-FU@FA-CHI-5-FAM are shown in Fig. 6. The luminescence spectra of ZIF-8/5-FU@FA-CHI-5-FAM are similar to those of 5-FAM, suggesting that the luminescent property of ZIF-8/5-FU@FA-CHI-5-FAM is mostly attributable to the emission of the 5-FAM. The emission spectra of 5-FAM, monitored under the characteristic excitation (437 nm) of the 5-FAM, exhibit a broad band with a maximum at 518 nm. Obviously, ZIF-8/5-FU@FA-CHI-5-FAM displays very strong emission with the maxima at 520 nm when excited at 441 nm, which is similar to the 5-FAM. These emission spectra indicate that the ZIF-8/5-FU@FA-CHI-5-FAM belongs to the class of luminescence materials with broad emission bands; in this case, the emission bands extend to the visible-light region and can already fulfill the requirements of bioimaging, as demonstrated in Fig. 7. For effective drug-based cancer treatment, the drug must be accumulated within tumours specifically. FA is considered one of the most promising targeting reagents for cancerous cells. Here, we evaluate the targeting efficacy of FA by incubating ZIF-8/5-FU@FA-CHI-5-FAM and ZIF-8/5-FU@CHI-5-FAM with FA-positive MGC-803 cells and FA-negative HASMC cells for 2 h. Our cellular uptake imaging in Fig. 7 clearly shows that the green fluorescence in MGC-803 cells incubated with ZIF-8/5-FU@FA-CHI-5-FAM is strong. By contrast, the green fluorescence in MGC-803 cells incubated with ZIF-8/5-FU@CHI-5-FAM and HASMC cells incubated with ZIF-8/5-FU@FA-CHI-5-FAM is very weak. In other words, ZIF-8/5-FU@FA-CHI-5-FAM (Fig. 7A) entered MGC-803 cells much more easily compared to free ZIF-8/5-FU@CHI-5-FAM (Fig. 7B). While, ZIF-8/5-FU@FA-CHI-5-FAM can hardly be taken on by the HASMC cells (Fig. 7C), indicating that ZIF-8/5-FU@FA-CHI-5-FAM is capable of cancer targeting.

**Figure 8:**
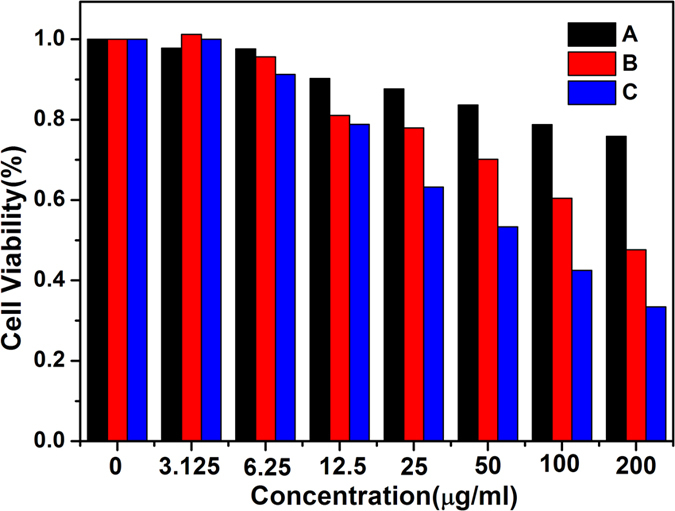
Viabilities of MGC-803 cells in the presence of ZIF-8 @ FA-CHI-5-FAM (**A**), ZIF-8/5-FU@FA-CHI-5-FAM (**B**) and 5-FU (**C**), as assayed by MTT.

*In vitro* cell viabilities of ZIF-8@ FA-CHI-5-FAM (Fig. 8A), ZIF-8/5-FU@FA-CHI-5-FAM (Fig. 8B) and free 5-FU (Fig. 8C) on MGC-803 cells are evaluated by MTT assay to study the bio-toxicity of ZIF-8@ FA-CHI-5-FAM and the therapeutic effect of ZIF-8/5-FU@FA-CHI-5-FAM. After 48 h incubation with MGC-803 cells, the MGC-803 cells treated with ZIF-8@ FA-CHI-5-FAM show no obvious toxicity when the concentration of the ZIF-8@FA-CHI-5-FAM is 200 μg/mL. However, ZIF-8/5-FU@FA-CHI-5-FAM exhibits a stronger cell growth inhibition effect on MGC-803 cells compared to ZIF-8@FA-CHI-5-FAM at the same concentration. Only 200 μg/mL of ZIF-8/5-FU@FA-CHI-5-FAM induces 55% death of the MGC-803 cells. Additionally, the cytotoxicity of ZIF-8/5-FU@FA-CHI-5-FAM is reduced against MGC-803 cells compared to the cytotoxicity of 5-FU alone, which is attributed to the lower amount of 5-FU contained in ZIF-8/5-FU@FA-CHI-5-FAM. Thus, we conclude that 5-FU can release from ZIF-8/5-FU@FA-CHI-5-FAM and induce cell death, which indicates that the ZIF-8/5-FU@FA-CHI-5-FAM can be potentially applied as an efficient drug-delivery system for cancer treatment.

**Figure 9:**
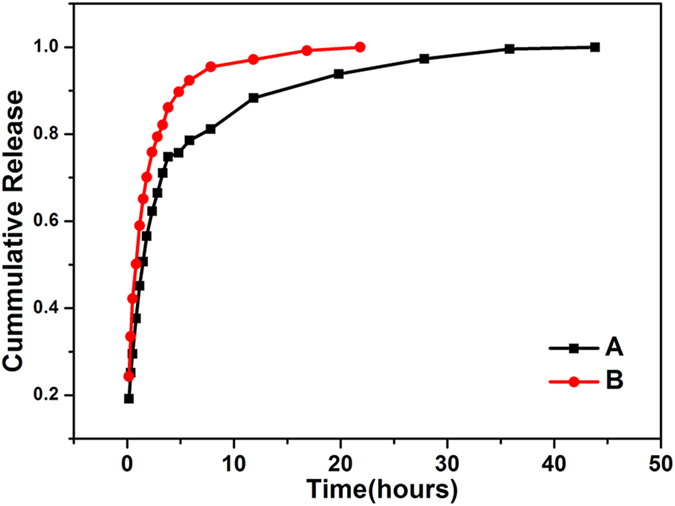
Drug release profiles for ZIF-8/5-FU@FA-CHI-5-FAM nanocrystals in PBS buffer solution at pH = 7.4 (**A**) and pH = 5 (**B**).

For the efficient and effective delivery of 5-FU, we designed a multifunctional hollow MOF that exhibits targeting ability and a high drug loading level. Here, the 5-FU encapsulation efficiency on ZIF-8/5-FU@FA-CHI-5-FAM is 51%, which is higher than the 32% encapsulation efficiency for the original ZIF-8. The large specific surface area of the hollow ZIF-8 is reasonably regarded as a cause of the high drug loadings. The BET surface area of the hollow ZIF-8 is as high as 1596 m^2^/g, whereas the BET surface area of solid ZIF-8 is only 136.7 m^2^/g (Supplementary Figures S9 and S10). Additionally, the controlled release of 5-FU from ZIF-8/5-FU@FA-CHI-5-FAM is conducted in PBS buffer solutions (pH = 7.4 and pH = 5) at 37 °C, and the drug delivery profiles are shown in Fig. 9. The concentration of 5-FU released from ZIF-8/5-FU@FA-CHI-5-FAM is obtained according to the standard equation. The UV-vis absorption spectra of 5-FU at different concentrations in PBS buffer solution and the standard 5-FU concentration curve are shown in Supplementary Figures S10 and S11, respectively. Through the curve fitting, we calculated the standard equation of 5-FU as A = 0.00186c + 0.06753, R^2^ = 0.99994, where A is the absorbance, c is the concentration of 5-FU solution and R is the correlation coefficient.

As a general observation, the release rate gradually decreases with time. The release of 5-FU can be divided into three phases: (1) an apparent initial burst release; (2) the gentle release phase and (3) the slow release phase. This initial rapid drug release is induced by the quick release of 5-FU molecules adsorbed onto the surface of ZIF-8/5-FU@FA-CHI-5-FAM. The other 5-FU molecules entrapped in the pores and cavities of ZIF-8/5-FU@FA-CHI-5-FAM are released slower. The release of 5-FU from ZIF-8/5-FU@FA-CHI-5-FAM in pH = 7.4 lasts for 45 h. In contrast, the 5-FU release rate is markedly increased in pH = 5 and this release reaches a plateau within 21 h, which is consistent with the dissolution of ZIF-8 and CHI in the acidic environment. Anyway, this result implies that the obtained ZIF-8/5-FU@FA-CHI-5-FAM exhibits a high drug loading level and a long sustained release time.

## Conclusion

In conclusion, a promising drug delivery system based on multifunctional hollow MOF (ZIF-8/5-FU@FA-CHI-5-FAM) is successfully developed for targeted tumour therapy and optical imaging. In this drug delivery system, the pores and hollow cavity of ZIF-8 can be used to load the anticancer drug 5-FU, FA modification of the shell provides the molecular targeting, and the imaging agent 5-FAM is used to monitor the drug delivery process through fluorescence imaging. Therefore, the as-synthesized ZIF-8/5-FU@FA-CHI-5-FAM exhibits excellent receptor-specific targeting effects for MGC-803 cells and shows an outstanding efficacy in killing the cancer cells. Additionally, the nature of the hollow ZIF-8/5-FU@FA-CHI-5-FAM for the storage and release of the 5-FU drug molecule is investigated; hollow ZIF-8 displays a higher drug loading level than the original ZIF-8. Under physiological conditions, the ZIF-8/5-FU@FA-CHI-5-FAM exhibits a sustained drug release for 45 h. Overall, this paper provides an efficacious method to explore MOFs as a new targeted drug delivery system, and we anticipate that this approach represents a promising platform for the efficient treatment of tumours. We are applying such materials as drug delivery systems, and an extension of this method is underway to address some broader biological applications.

## Experimental Section

### Material and Methods

All the starting reagents and solvents were of A.R. grade and were acquired from commercial sources; they were used directly without further purification. Power X-ray diffraction (PXRD) patterns were collected on a PANalytical Empyrean sharp shadow system X-ray diffractometer at a scanning rate of 2°/min in the 2θ range from 5° to 80°; the diffractometer was equipped with a Cu Kα radiation source (λ = 1.540598 Å). The size and morphology of the as-prepared nanocrystals were investigated using a HITACHI S-4800 200 kV scanning electron microscope (SEM). Transmission electron microscopy (TEM) was performed using a FEI Tecnai G^2^ F20 high-resolution transmission electron microscope. Energy-dispersive X-ray (EDX) mapping analysis was carried out on a scanning electron microscope equipped with an EDX apparatus. Photoluminescence (PL) spectra were collected on a Hitachi F-7000 fluorescence spectrophotometer. Fourier transform infrared spectroscopy (FTIR) spectra were recorded with a NEXUS-670 Fourier transform infrared spectrophotometer. Thermogravimetric analysis (TGA) was performed from 40 °C to 600 °C at a heating rate of 10 °C/min using a NETZSCH STA449F3 thermal analyser; the samples were maintained under an N_2_ atmosphere during the analysis. A laser scanning confocal microscope (OLYMPUS, IX81) was used to analyse the cell proliferation and the distribution of drug carriers. UV-Vis spectra were recorded in the wavelength range from 200 to 500 nm at room temperature using a TU-1901 diode UV-visible spectrophotometer.

### Construction of hollow ZIF-8

The hollow ZIF-8s with excellent crystalline structures were obtained via a mild phase transformation under solvothermal conditions, as previously reported^34^. In a normal procedure, 0.558 g Zn(NO_3_)_2_ · 6H_2_O was dissolved in 15 mL methanol to obtain solution A and 0.616 g 2-methylimidazole was dissolved in 15 mL methanol to obtain solution B. After forming a homogeneous solution, solution A was added dropwise to solution B, followed by ultrasonication for 15 min at room temperature. The product was then separated via centrifugation at 11,000 rpm twice and redispersed in 15 mL methanol by ultrasonication to form solution C. Subsequently, 0.558 g Zn(NO_3_)_2_ · 6H_2_O in 15 mL methanol solution was added to solution C and then transferred to 50 mL Teflon-lined stainless steel autoclaves. The mixture was then hydrothermally treated at 120 °C for 2 h. Finally, the mixture was washed via centrifugation with methanol several times and dried at 30 °C in a vacuum oven.

### Preparation of FA-CHI-5-FAM

The FA-CHI-5-FAM conjugates were prepared through a dehydration condensation reaction between the carboxyl groups of the FA, 5-FAM and amino groups of the CHI chain. Briefly, 10 mg FA and 1 mg 5-FAM were dissolved in 50 mL dimethyl sulfoxide (DMSO) with stirring to form solution A. Then, 10 mg CHI in an acetic acid aqueous solution (0.1 M, pH 4.7) was mixed with solution A to product solution B under continuous stirring. Afterwards, 20 mg N-(3-dimethylaminopropyl)-N -ethylcarbodiimide hydrochloride (EDC) was added to solution B, followed by stirring in the dark at room temperature for 16 h to allow the FA and 5-FAM to conjugate onto the CHI molecules. The solution was brought to pH 9.0 with NaOH aqueous solution (1.0 M), and the purified FA-CHI-5-FAM conjugate was obtained by centrifugation, washed with DMSO several times and finally freeze-dried.

The aforementioned synthetic procedure was subsequently used to synthesize CHI-5-FAM. Details of the procedure are provided in the supporting information.

### Preparation of ZIF-8/5-FU@FA-CHI-5-FAM

Typically, 0.1 g hollow ZIF-8, 0.1 g of 5-FU and 0.5 g FA-CHI-5-FAM were added to deionized water under ultrasound, and the solution was then stirred for 4 days at room temperature. Subsequently, the product was collected by centrifugation, washed with distilled water two or three times, and then dried under vacuum at 25 °C.

The aforementioned synthetic procedure was used to synthesize ZIF-8/5-FU@CHI-5-FAM and ZIF-8@FA-CHI-5-FAM. The detailed procedure is described in the supporting information.

### Loading efficiency of 5-FU

To determine the encapsulation efficiency, several 5-FU water solutions in the 0–10 μg/mL concentration range were prepared to obtain a calibration curve. The amount of 5-FU not loaded but contained in the excess of solvent was measured by UV-V is absorption at its wavelength of maximum absorbance (λmax = 265 nm) and subsequently calculated on the basis of the calibration curve. The drug loading efficiency was calculated by the following equation: loading efficiency (%) = (m_1_ − m_2_)/m, where m_1_, m_2_ and m represent the initial weight of 5-FU, the weight of 5-FU present in the excess of solvent and the weight of the hollow ZIF-8, respectively. After 4 days of soaking, the loading efficiency of 5-FU on hollow ZIF-8 was 51%, which is greater than the 32% loading efficiency of 5-FU on solid ZIF-8.

### Cytotoxicity study

The *in vitro* cytotoxicities of ZIF-8@FA-CHI-5-FAM, ZIF-8/5-FU@FA-CHI-5-FAM and 5-FU were assessed in MGC-803 cells by the MTT method. In brief, MGC-803 cells were added to each well of a 96-well plate and incubated for 24 h. Subsequently, different concentrations of ZIF-8@FA-CHI-5-FAM, ZIF-8/5-FU@FA-CHI-5-FAM and 5-FU (0, 6.25, 12.5, 25, 50 and 100 μg/mL) were added to the wells and incubated for another 48 h. After the previous nutrient solution was removed, 20 μL of MTT solution was added to each well and incubated for another 4 h. All media were then removed, and 100 μL of DMSO was added to each well. The absorbance of each sample was monitored at 570 nm using a microplate reader. The cell viability was expressed as a percentage of the absorbance of the sample well to that of the cell control. All experiments were performed in triplicate, and the results were averaged.

### Cellular uptake study

MGC-803 cells and HASMC cells were seeded into a 6-well plate. After culturing for 24 h at 37 °C, the cells were washed three times with phosphate-buffered saline (PBS) and blocked in PBS containing BSA (1%) at 4 °C for 20 min. The cells were then incubated with ZIF-8/5-FU@FA-CHI-5-FAM and ZIF-8/5-FU@CHI-5-FAM for 2 h (concentration was 0.1 μg/mL). After three washings with PBS buffer, cell targeting was detected with a laser scanning confocal microscope (OLYMPUS, IX81). Cell targeting was detected on a laser scanning confocal microscope for luminescence imaging under excitation wavelength of 405 nm.

### *In vitro* drug release study

The release assays were carried out by soaking the samples in phosphate-buffered saline (PBS, pH 7.4) at 37 °C. In short, 0.05 g ZIF-8/5-FU@FA-CHI-5-FAM was introduced into a dialysis bag and then immersed into 10 mL PBS solution in a 50 mL centrifuge tube at 37 °C. At predetermined time intervals, 2 mL of solution was withdrawn, and the amount of 5-FU released from the ZIF-8/5-FU@FA-CHI-5-FAM was examined at 265 nm by recording the UV absorbance of the solution. The cumulative release percentages of 5-FU from ZIF-8/5-FU@FA-CHI-5-FAM were calculated as follows and plotted against time: Cumulative 5-FU release = amount of released 5-FU/amount of total 5-FU × 100%.

## Additional Information

**How to cite this article**: Gao, X. *et al.* Fabrication of functional hollow microspheres constructed from MOF shells: Promising drug delivery systems with high loading capacity and targeted transport. *Sci. Rep.* **6**, 37705; doi: 10.1038/srep37705 (2016).

**Publisher's note:** Springer Nature remains neutral with regard to jurisdictional claims in published maps and institutional affiliations.

## Supplementary Material

Supplementary Information

## Figures and Tables

**Figure 1:**
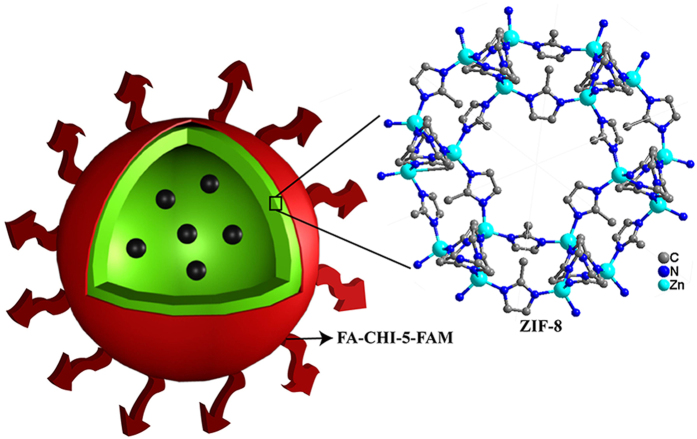
Schematic of ZIF-8/5-FU@FA-CHI-5-FAM nanocomposites.
