# Supplementary material for: Fabrication of functional hollow microspheres constructed from MOF shells: Promising drug delivery systems with high loading capacity and targeted transport
Source: Sci Rep. 2016 Nov 23;6:37705. doi: 10.1038/srep37705 (PMC5120282; doi:10.1038/srep37705)
Supplement: Supplementary Information [file srep37705-s1.doc]

**Electronic Supporting Information for**

**Fabrication of functional hollow microspheres constructed from MOF shells: Promising drug delivery systems with high loading capacity and targeted transport**

**Xuechuan Gao, Xiao Hai, Huricha Baigude, Weihua Guan, and Zhiliang Liu***

College of Chemistry and Chemical Engineering, Inner Mongolia University, Hohhot, 010021, P. R. China. E-mail: cezlliu@imu.edu.cn; Fax: +86-471-4994375; Tel: +86-18686029088

Corresponding author.

Tel: +86-471-4994375; Fax: 0086-471-4994375

E-mail addresses: cezlliu@imu.edu.cn (Z. L. Liu)

**Supplementary Information:**

**Experimental section**

**Figure S1. TEM images of solid ZIF-8 with different magnificatios (A,B)**

**Figure S2.** [**Fluorescence**](../../../../C:/Users/Administrator/AppData/Local/Yodao/DeskDict/frame/20160103093506/javascript:void(0)%3B) [**imaging**](../../../../C:/Users/Administrator/AppData/Local/Yodao/DeskDict/frame/20160103093506/javascript:void(0)%3B) **of ZIF-8/5-FU@FA-CHI-5-FAM detected on a laser scanning confocal microscope under excitation wavelength of 405 nm.**

**Figure S3. Size distribution of ZIF-8(A) and ZIF-8/5-FU@FA-CHI-5-FAM (B)**

**Figure S4. Chemical structure of FA-CHI-5-FAM**

**Figure S5. Uv-vis absorption spectra of 5-FAM (A), CHI (B), FA (C) and FA-CHI-5-FAM (D).**

**Figure S6. TGA curves of hollow ZIF-8(A) and ZIF-8/5-FU@FA-CHI-5-FAM (B)**

**Figure S7. The absorbance of FA in aqueous solution as a function of concentration (A) and the calibration curve of A (B)**

**Figure S8. The absorbance of 5-FAM in aqueous solution as a function of concentration (A) and the calibration curve of A (B)**

**Figure S9. N_2_ adsorption-desorption isotherms and pore size distribution (the insert) of hollow ZIF-8. The adsorption branch is shown in black color (A) and the desorption branch in red color (B)**

**Figure S10. N_2_ adsorption-desorption isotherms and pore size distribution (the insert) of solid ZIF-8 spheres. The adsorption branch is shown in black color (A) and the desorption branch in red color (B)**

**Figure S11. UV-vis absorption spectra of 5-FU at different concentrations in PBS buffer solution: A (0 µg/mL), B (2 µg/mL), C (4 µg/mL), D (6 µg/mL), E (8 µg/mL), F (10 µg/mL)**

**Figure S12. The absorbance of 5-FU in PBS buffer solution as a function of concentration (A) and the calibration curve of A (B)**

**Experimental Section**

**Preparation of CHI-5-FAM**

CHI-5-FAM conjugates could be prepared through a dehydration condensation reaction between the carboxyl groups of 5-FAM and amino groups of CHI chain. Shortly, 1mg 5-FAM was dissolved in 50 mL dimethyl sulfoxide (DMSO) with stirring to form soultion A. Then 10 mg CHI in acetic acid aqueous solution (0.1 M, pH 4.7) was mixed with soultion A to product soultion B under continuous stirring. After that, 20 mg N-(3-dimethylaminopropyl)-N-ethylcarbodiimide hydrochloride (EDC) was added into soultion B, followed by stirring in the dark at room temperature for 16 h to let 5-FAM conjugate onto CHI molecules. The solution was brought to pH 9.0 with NaOH aqueous solution (1.0 M) and the CHI-5-FAM conjugate purified was obtained by centrifugation and washed with DMSO several times and finally freeze-dried.

**Preparation of ZIF-8/5-FU@CHI- 5-FAM**

Typically, 0.1 g hollow ZIF-8, 0.1 g of 5-FU and 0.5 g CHI-5-FAM were added into deionized water under ultrasound and then stirring the solution for 4 days at room temperature. Subsequently, the product was collected by centrifugation, washed with distilled water for three times, and then dried in vacuum at 25 °C.

**Preparation of ZIF-8@FA-CHI- 5-FAM**

Typically, 0.1g hollow ZIF-8 and 0.5g FA-CHI-5-FAM were added into deionized water under ultrasound and then stirring the solution for 48 h at room temperature. Subsequently, the product was collected by centrifugation, washed with distilled water for two or three times, and then dried in vacuum at 25 °C.


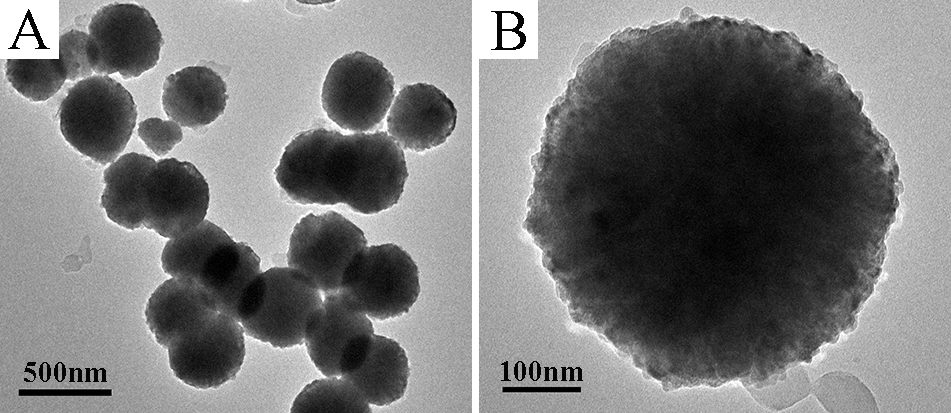


Figure S1. TEM images of solid ZIF-8 with different magnificatios (A, B)


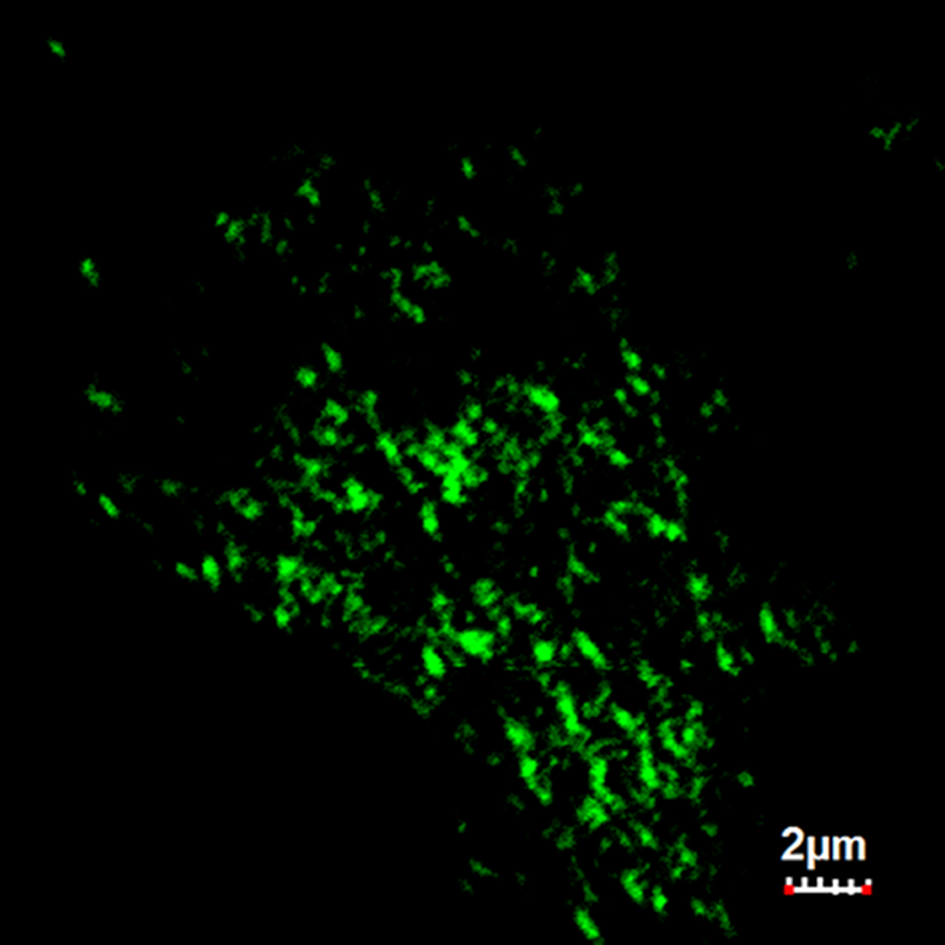


Figure S2. [Fluorescence](../../../../C:/Users/Administrator/AppData/Local/Yodao/DeskDict/frame/20160103093506/javascript:void(0)%3B) [imaging](../../../../C:/Users/Administrator/AppData/Local/Yodao/DeskDict/frame/20160103093506/javascript:void(0)%3B) of ZIF-8/5-FU@FA-CHI-5-FAM detected on a laser scanning confocal microscope under excitation wavelength of 405 nm.





Figure S3. Size distribution of ZIF-8(A) and ZIF-8/5-FU@FA-CHI-5-FAM (B)


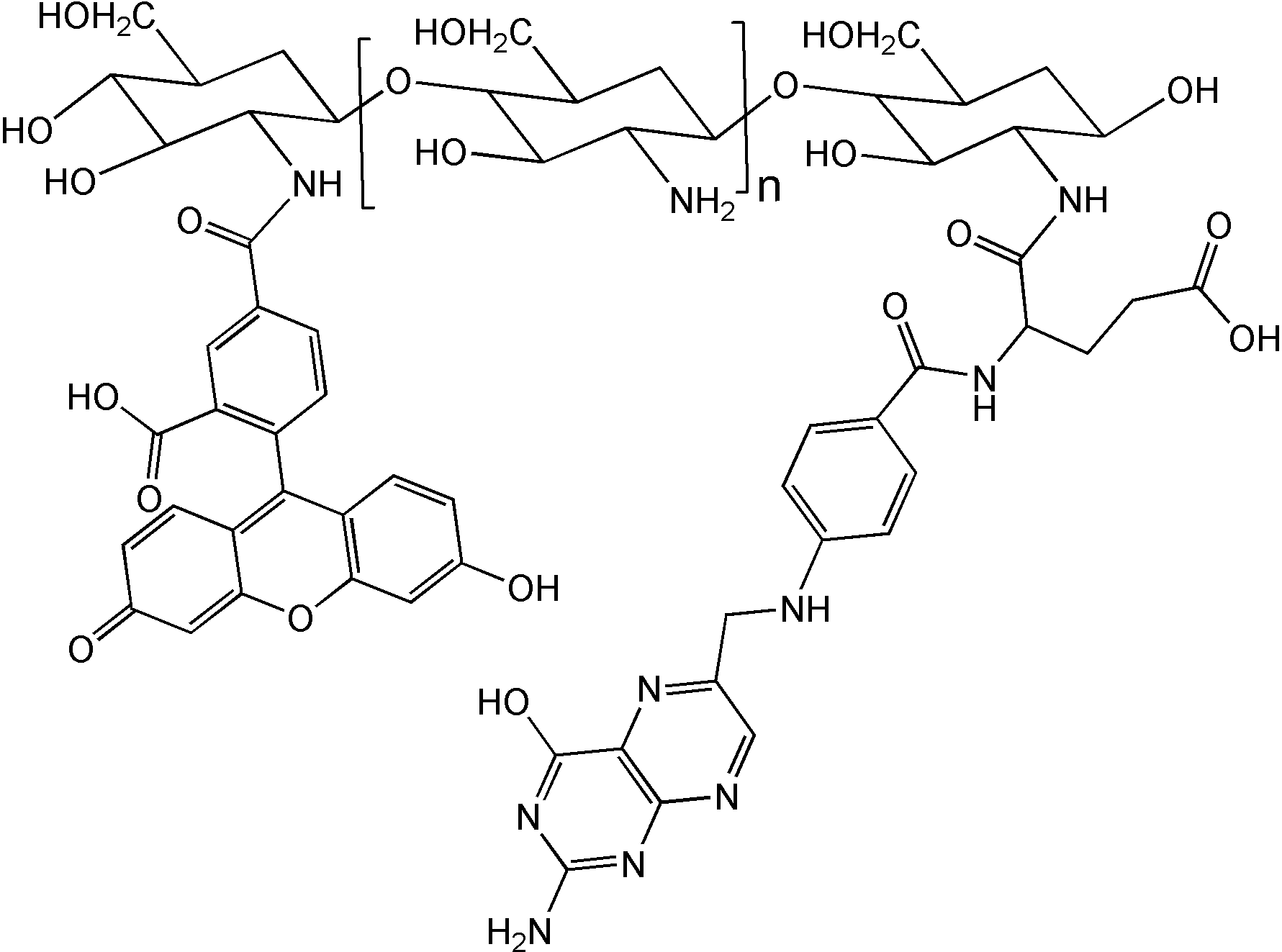


Figure S4.Chemical structure of FA-CHI-5-FAM


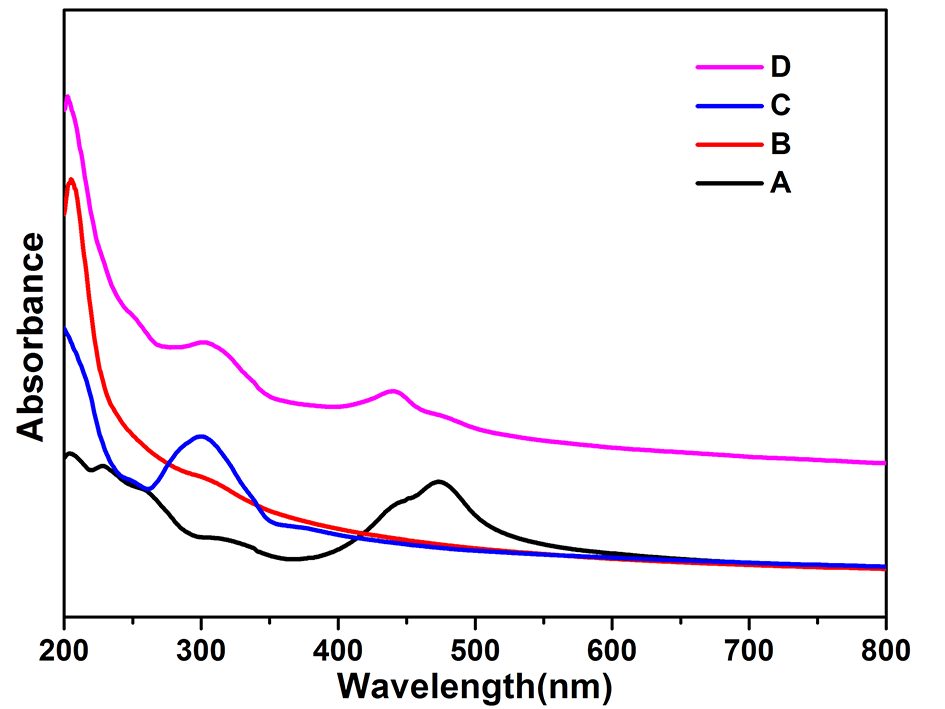


Figure S5. Uv-vis absorption spectra of 5-FAM (A), CHI (B), FA (C) and FA-CHI-5-FAM (D).


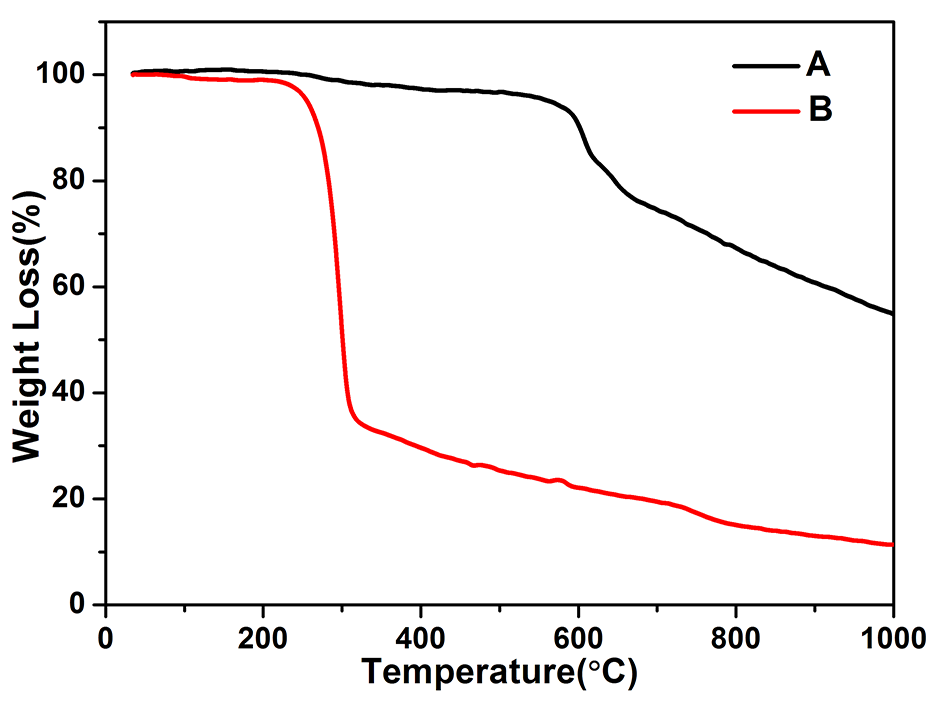


Figure S6. TGA curves of hollow ZIF-8(A) and ZIF-8/5-FU@FA-CHI-5-FAM (B)


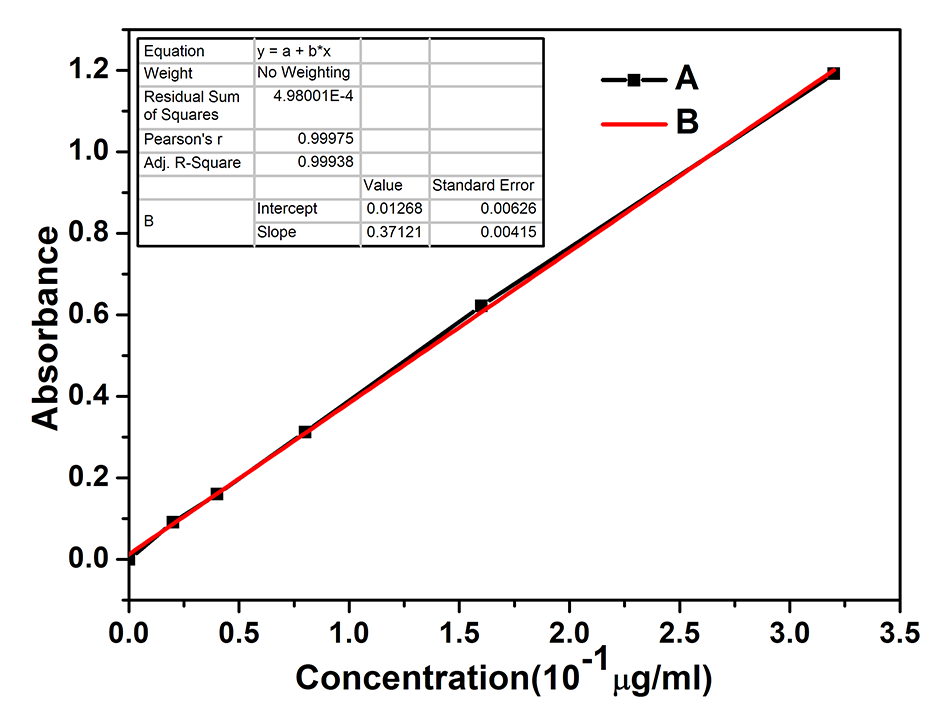


Figure S7. The absorbance of FA in aqueous solution as a function of concentration (A) and the calibration curve of A (B)





Figure S8. The absorbance of 5-FAM in aqueous solution as a function of concentration (A) and the calibration curve of A (B)


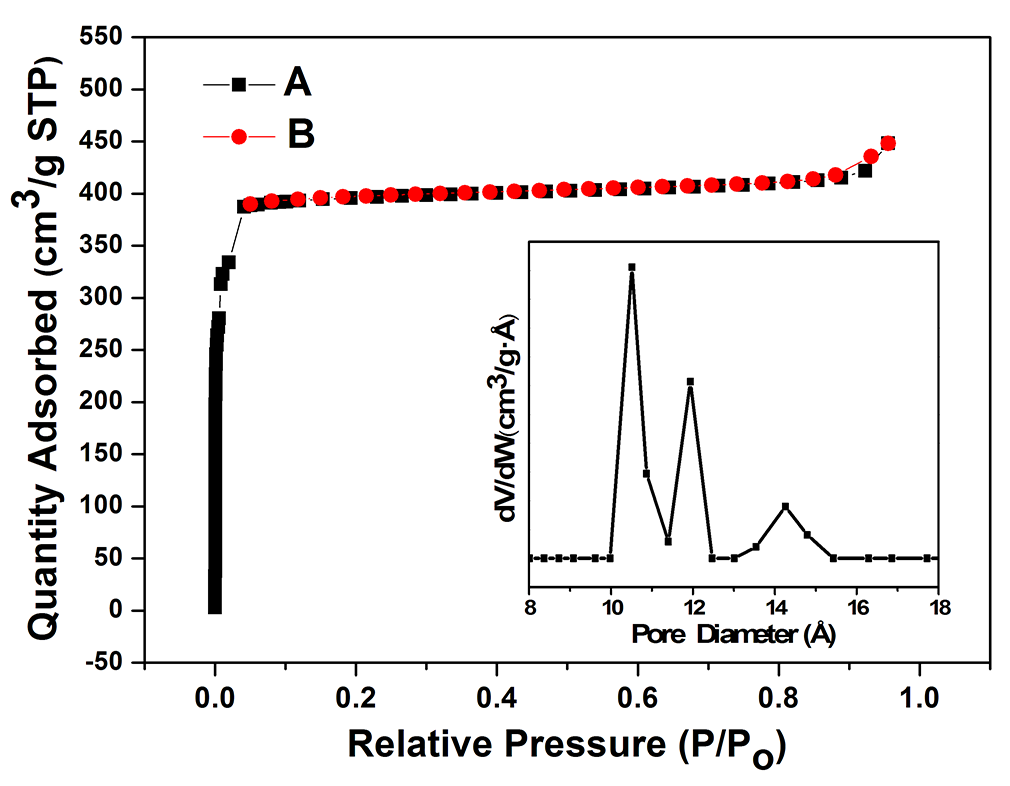


Figure S9. N_2_ adsorption-desorption isotherms and pore size distribution (the insert) of hollow ZIF-8. The adsorption branch is shown in black color (A) and the desorption branch in red color (B)


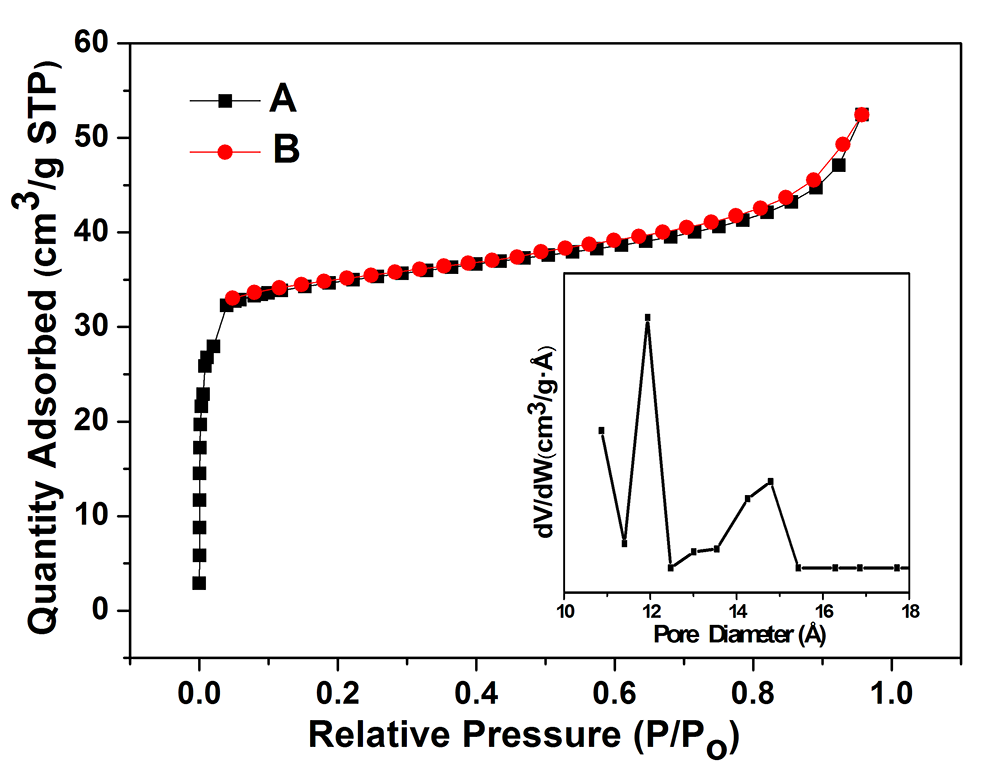


Figure S10. N_2_ adsorption-desorption isotherms and pore size distribution (the insert) of solid ZIF-8 spheres. The adsorption branch is shown in black color (A) and the desorption branch in red color (B)


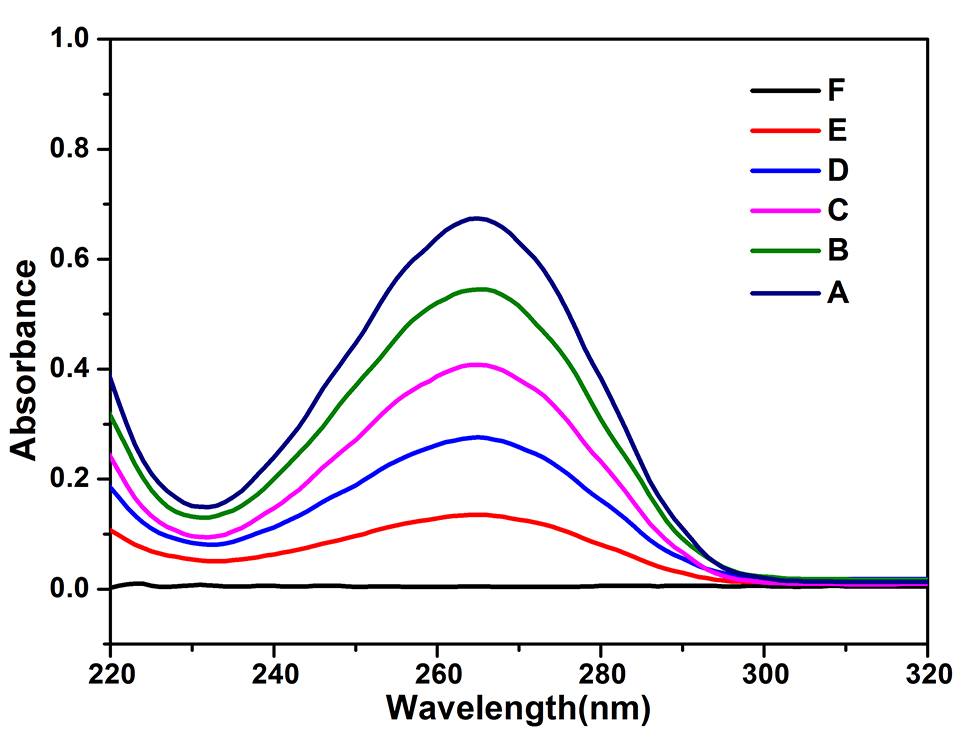


Figure S11. UV-Vis absorption spectra of 5-FU at different concentrations in PBS buffer solution: A (0 µg/mL), B (2 µg/mL), C (4 µg/mL), D (6 µg/mL), E (8 µg/mL), F (10 µg/mL)


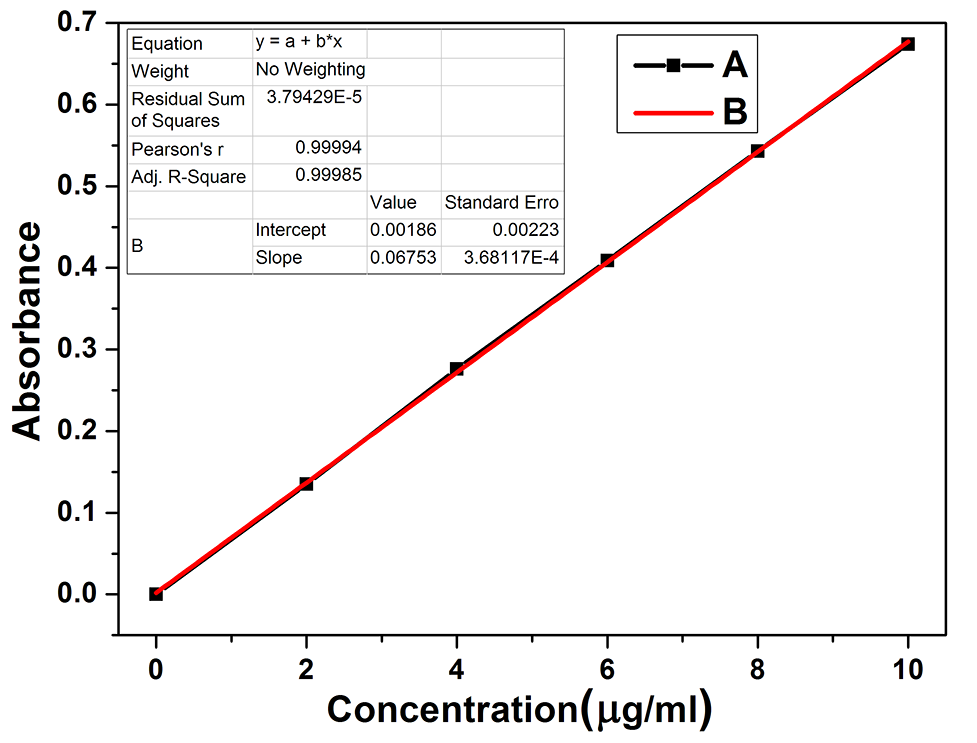


Figure S12. The absorbance of 5-FU in PBS buffer solution as a function of concentration (A) and the calibration curve of A (B)
